# Supplementary material for: Intermolecular channels direct crystal orientation in mineralized collagen
Source: Nat Commun. 2020 Oct 8;11:5068. doi: 10.1038/s41467-020-18846-2 (PMC7545172; doi:10.1038/s41467-020-18846-2)
Supplement: Supplementary file 1 — Supplementary Information [file 41467_2020_18846_MOESM1_ESM.pdf]

Supplementary Information for

Intermolecular Channels Direct Crystal Orientation in Mineralized Collagen

Xu et al.

Supplementary Figures

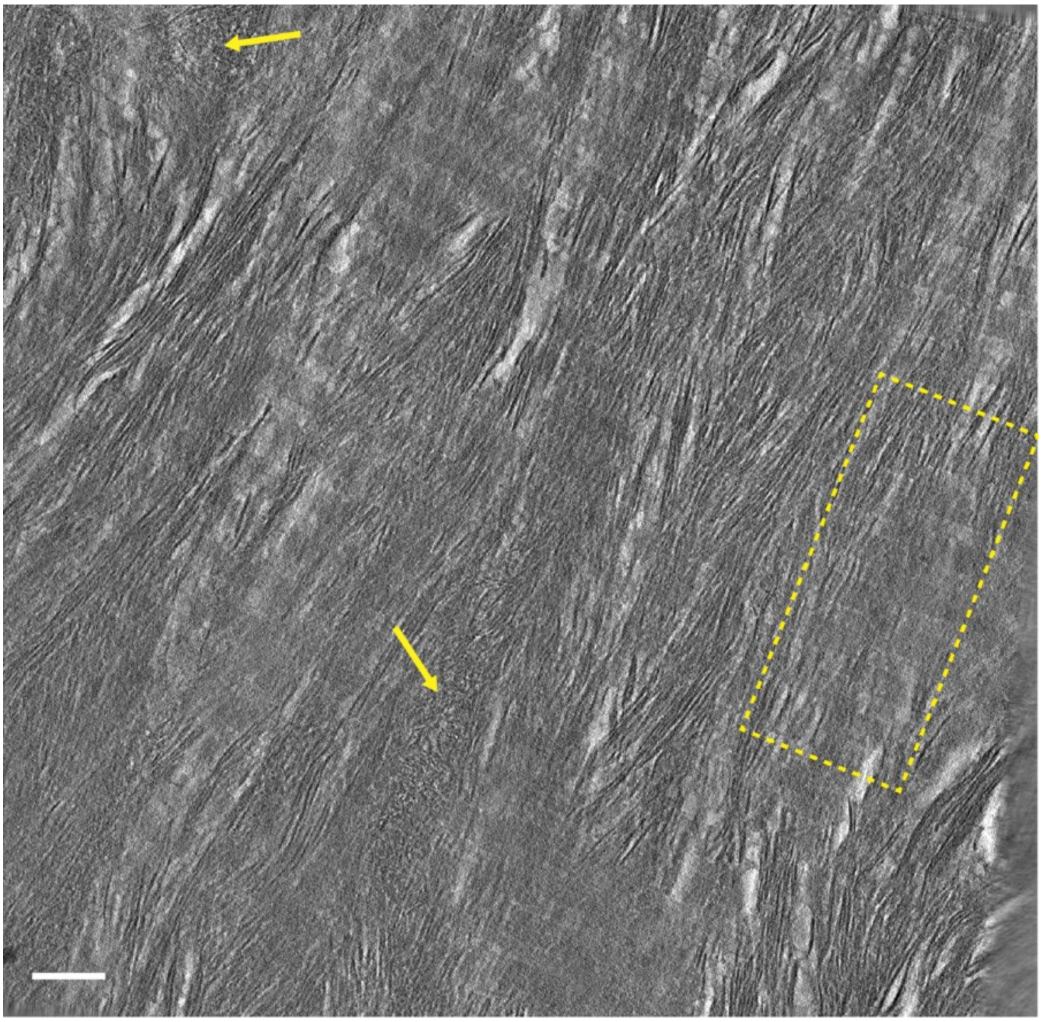

Figure 1. 2D projection of tomographic reconstruction slices (70 y-slices averaged), showing an overview of the human bone lamella. Scale bar: 100 nm.

Supplementary Figure 1 is a 2D projection of tomographic reconstruction y-slices (70 y-slices averaged), showing an overview of the human bone lamella. Most of the area shows a clear ~67 nm banding pattern, indicating that collagen fibrils are closely packed, and their long axes are generally in plane. Gap regions are darker than the overlap regions due to heavy metal staining of the sample (using the OTOTO method, similar to Landis using uranyl acetate[1,2]), not due to mineralization, as no higher mineral density is observed. Indeed, the in vitro HAp/collagen (Figure 2i), which was only lightly stained, does not show this an effect. In some areas (highlighted by yellow arrows) the banding pattern is less clear, indicating that the long axes of collagen fibrils within these areas are tilted out of plane, as a result of the 3D twisting of collagen fibrils. The area in the yellow box is selected for analysis in Figure 2 in the main text, as a clear discontinuity can be seen between the two adjacent collagen fibrils within this area, which helps to identify the boundary between the two fibrils.

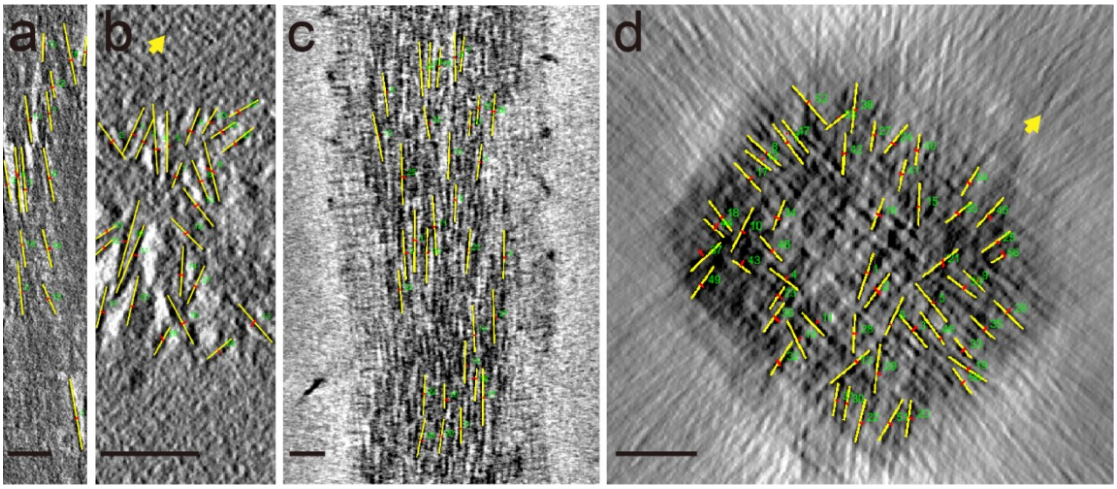

Figure 2. Size analysis on the tomographic reconstruction slices, in which the length of long (yellow lines) and short axes (red lines) of the crystals are determined. (a,b) Mineralized collagen fibril in human bone viewed from the top (y-slice) and along the fibril (z-slice), respectively (c,d) collagen fibril mineralized in vitro by HAp viewed from the top (y-slice) and along the fibril (z-slice), respectively. Dark streaks are artifacts induced by limitation of tilt conditions and are highlighted by yellow arrows in b) and d). Scale bars: 50 nm

To determine the dimensions of the crystallites that were grown within the collagen a size analysis was performed. To that end tomographic slices were loaded as images into Matlab and the long and short axis were manually determined by clicking the four edges of the crystallites. The slices from the tomogram were taken such that the crystallites were at least 10 nm (in y-direction) or 50 nm (in z-direction) apart so the same crystals are not measured more than once. The contrast of the z-slices was enhanced by averaging 10 adjacent slices. A sample of the size analysis that was performed on different sections for both the mineralized collagen fibril from human bone and the in vitro mineralized collagen fibril is shown in Supplementary Figure 2. Dark streaks are visible in the z-slices (as pointed out by yellow arrows in Supplementary Figures 2b and 2d) due the limited tilt increment (2° per step) and tilt range (−65 to 65°) used for collecting the tomography tilt series.[3] These streaks are however low in contrast and each of them only exist within several slices (see Supplementary Movies 3 and 5), while a HAp crystal will propagate through ~85 z-slices due its length (~65 nm). This allows to confidently distinguish between the streaks and HAp crystals.

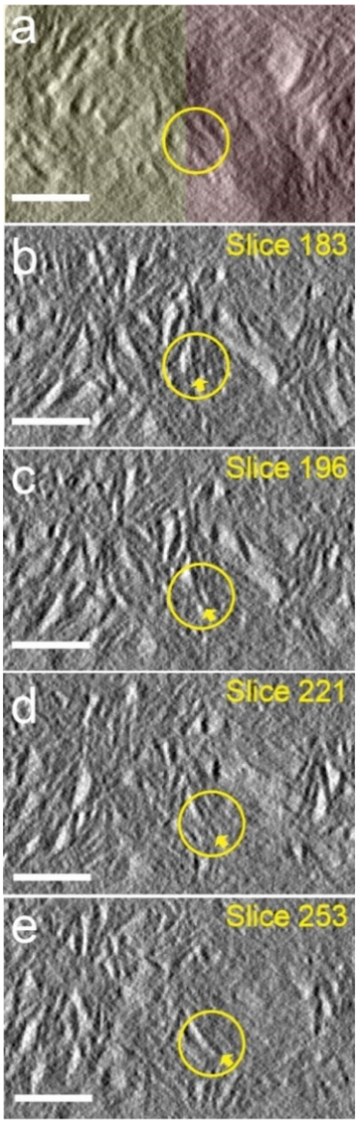

Figure 3. (a) 2D projection of 400 cross section tomographic reconstruction z-slices (total thickness 300 nm) showing two adjacent fibrils, as highlighted by yellow and red colors, respectively. (b-e) Cross section z-slices of the two fibrils with slice number 183, 196, 221 and 253, respectively. Each slice is averaged from 10 adjacent slices to enhance the contrast. The cross sections of one same HAp platelet in the four slices are identified by yellow circles, while the lateral orientation of the platelet is highlighted by yellow arrows. The position of yellow circle in (b) is also highlighted in (a). Scale bars: 50 nm.

Supplementary Figure 3 tracks one HAP platelet through different cross section z-slices of two adjacent collagen fibrils (as shown in Figs. 2a and 2e). The distance between Supplementary Figures 3b (slice 183) and 3e (slice 253) is 52.5 nm (70 slices, slice thickness=0.75 nm). The platelet extends from the boundary between the two fibrils into the fibril on the right side. Note that the lateral orientation of the platelet has rotated counter clockwise with ~30° between Supplementary Figures 3b and 3e, indicating that the platelet is twisted like a propeller as previously reported.[4] Such twisting can also be observed for mineral platelets that are completely within the fibrils.

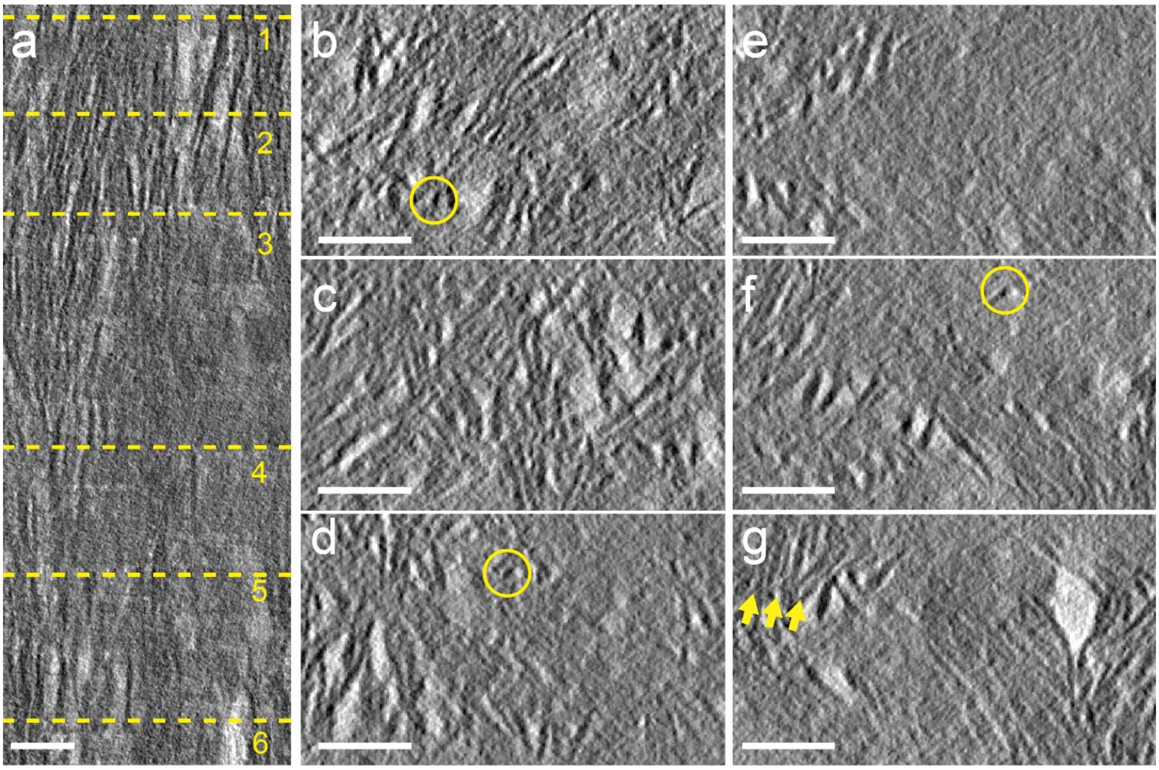

Figure 4. (a) 2D projection of the top-view tomographic reconstruction y-slices showing two adjacent mineralized collagen fibrils in human bone. (b-g) Cross section reconstruction z-slices at positions 1-6, respectively, as labelled in (a). Each slice is averaged from 10 adjacent slices to enhance the contrast. Several dots correspond to the needle-shaped tips of the mineral platelets are highlighted by yellow circles, while several stacks consist of ~8 platelets are highlight in (g) by array of yellow arrows. Scale bars: 50 nm.

Supplementary Figure 4 shows 6 cross section tomography reconstruction z-slices selected at different regions of the two adjacent fibrils. Randomly oriented stacks of 2-4 HAp platelets can be seen in most of the slices, while in the last slice (Supplementary Figure 4g) there are several larger stacks of ~8 platelets. These stacks are ~50 nm in size and have different lateral orientations. The dots highlighted by yellow circles correspond to the needles found at the tip of platelets.

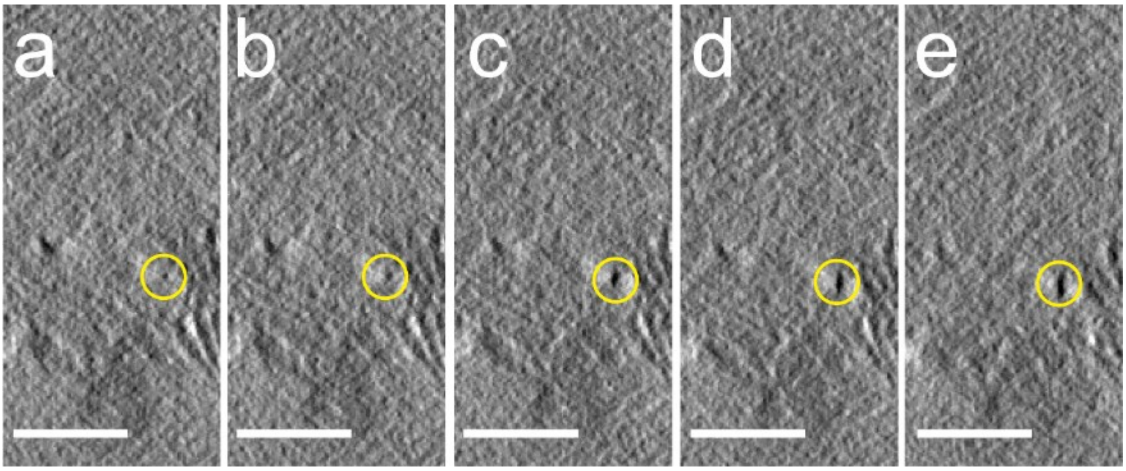

Figure 5. (a-e) Cross section z-slices of one collagen fibril with slice number 579, 574, 568, 566 and 553, respectively. Each slice is averaged from 10 adjacent slices to enhance the contrast. The cross-sections of the needle which evolves into platelet are highlighted by yellow circles. Scale bars: 50 nm.

By tracking a dot in different cross section z-slices (Supplementary Figure 5), it is clear that the dot actually corresponds to a needle which is ~2 nm in width. The needle evolves into a platelet-like shape in Supplementary Figure 5d, suggesting that its length is about ~10 nm (spanning 14 z-slices, slice thickness 0.75 nm), and the needle is actually the tip of a HAp platelet.

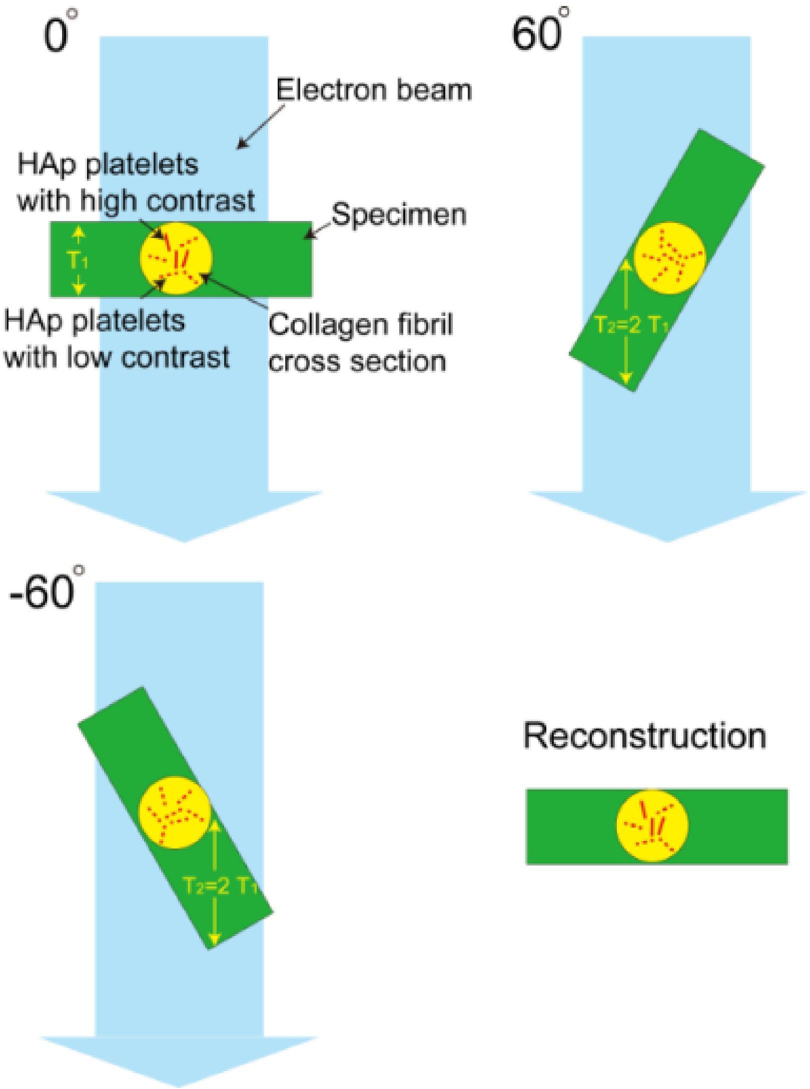

Figure 6. Contrast of HAp platelets in collagen fibrils at different tilting angles. At low tilting angle, e.g., 0°, the edge-on platelets are more visible, while the platelets in other orientations are low in contrast. At high tilting degrees, e.g., ±60°, however, all the platelets are lower in contrast as the actual sample thickness T_2 has doubled comparing with the thickness at 0°. As a result, the platelets that are edge-on at low tilting angles are more visible in the final reconstruction.

As our results contrast the early results of Landis et al. on turkey tendon[2] and chick bone,[1] we discuss here the origin of these differences, and propose that they originate from a difference in sample preparation and visualization. As shown in Supplementary Figure 6, the tomography samples consist of mineralized collagen fibrils aligned within a ~100 nm slab of epoxy-embedded bone material. At low tilting angle, e.g., 0°, the platelets with edge-on orientation are high in contrast due to their widths (~20 nm as we observed), while the platelets in other orientations (non-edge-on platelets) are less visible as they are very thin (2~4 nm). At higher tilting angles, e. g., 60°, the projected sample thickness T_2 is doubled with respect to the projected thickness at 0° (T_1). This reduces the contrast of all platelets with respect to the matrix at higher tilt angles, even more so when the sample is relatively thick. Therefore, the non-edge-on platelets that would to be more visible at these higher tilt angles are still low in contrast. As a result, the parallel organized platelets that are edge-on at low tilting angles will be significantly more visible in the final reconstruction than the non-edge-on platelets, thereby generating the impression of a deck of cards organization.

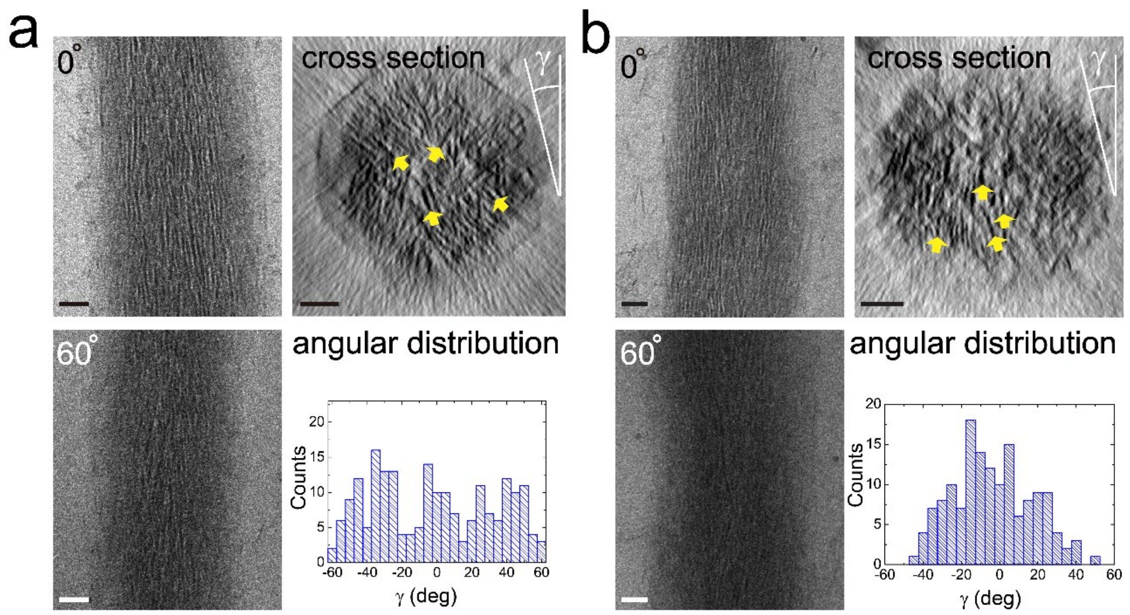

Figure 7. Sample thickness effect for cryo-electron tomography observation of collagen fibrils mineralized by HAp in vitro. (a) In a thinner area, edge-on HAp platelets are visible at both 0° and 60° tilting angles. In this case, no lateral orientation was observed for the platelets in the final reconstruction. (b) In a thicker area, edge-on platelets are still visible when tilting angle=0°. At 60°, however, the overall image contrast is very low, with the platelets hardly visible. As a result, in the final reconstruction, the platelets show a pseudo lateral orientation, with the angular distribution center at 0°. Lateral orientation of several HAp platelets are highlighted by yellow arrows Scale bars: 50 nm.

We can emulate this effect in our cryo-electron tomography study of the in vitro mineralized collagen fibril. For the same sample, in the area where ice layer is relatively thin (Supplementary Figure 7a, also Figures 2i to 2p in the main text), edge-on HAp platelets are observed at tilt angles of both 0° and 60°, although the image contrast is lower at 60°. In this case, a random lateral orientation was observed for these platelets in the final reconstruction. In a thicker area (Supplementary Figure 7b), however, the image contrast at a tilt angle of 60° is very low. In the reconstruction, a pseudo lateral orientation was observed for the HAp platelets, with the angular distribution centered at 0°.

In the 1993/1996 work of Landis et al. on tendon/chick bone,[1,2] the samples were ~500/~250 nm thick sections, which are both significantly thicker the sample used in our study (~100 nm thick). The TEM used for the previous two studies was a 1.0 MV Albany AEI-EM7 high voltage electron microscope, and the images were recorded using an EICONIX EC 78/99 digital camera with a pixel size of 1.3 nm. Although that the Albany AEI-EM7 has a higher accelerating voltage, the resolution/contrast achieved is unlikely to compete with the more recent FEI-Titan 300 kV TEM with large pole gap and energy filter that was used in our study, as the resolution in TEM is determined by many factors including the quality of electron source and lens, stability of stage, sensitivity of camera, use of energy filter, etc. Especially, at higher acceleration voltages the electron scattering cross-section of the specimen will be lower, which is actually not favored for beam-sensitive specimen with light elements (e.g., bone).[5] Indeed, in the 1996 work of Landis et al.,[1] the thickness of HAp platelets in bone was observed to be as high as 8 nm, suggesting a lack of resolution as a 2~4 nm thickness was observed in most of the recent studies.

The above shows that the intrafibrillar HAp platelets in bone are only uniaxially oriented, and explains that in the mid 1990's the state of the technology was not such that it could be observed in the pioneering experiments of Landis et al.

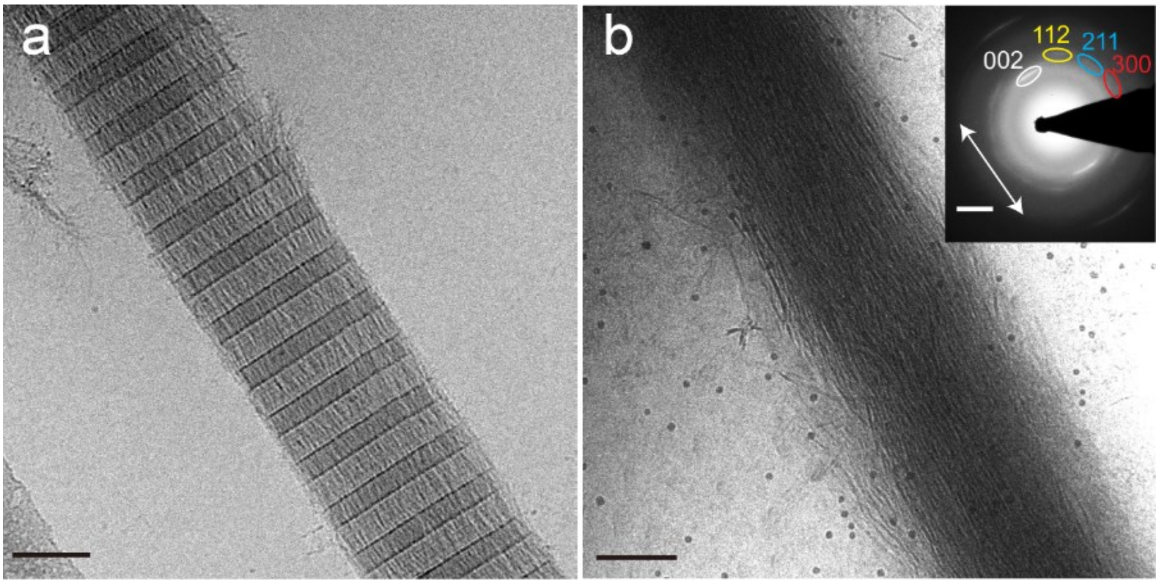

Figure 8. CryoTEM image of collagen fibrils (bovine Achilles) (a) before and (b) after HAp mineralization. Inset of (b): LDSAED pattern, with diffractions corresponding to HAp highlighted. The arrow indicates the direction of the collagen fibril. Scale bars: (a,b) 100 nm. Inset of (b): 2 nm^{-1}.

Type-I collagen fibrils separated from collagen sponge (bovine Achilles) by grinding in liquid nitrogen and re-disperse in HEPES buffer were visualized by cryoTEM (Supplementary Figure 8a), which shows ~300 nm thick fibrils with a clear 67 nm banding pattern, identical to what has been observed for self-assembled type I collagen fibrils (Horse Tendon).[6] The fibrils were mineralized by being exposed to a reaction solution containing $CaCl_2$, K_2HPO_4 and poly(aspartic acid) (pAsp). Significant intrafibrilar HAp mineralization was observed after 4 days (Supplementary Figure 8b). LDSAED (inset of Supplementary Figure 8b) shows a pair of narrow arcs corresponding to HAp (002) planes in the direction of collagen fibrils, together with 3 pairs of arcs with similar d-values corresponding to the (112), (221) and (300) diffractions, respectively. This observations is same with HAp mineralized horse tendon collagen (Figures 2i and 2l), and in-line with a previous report.[7]

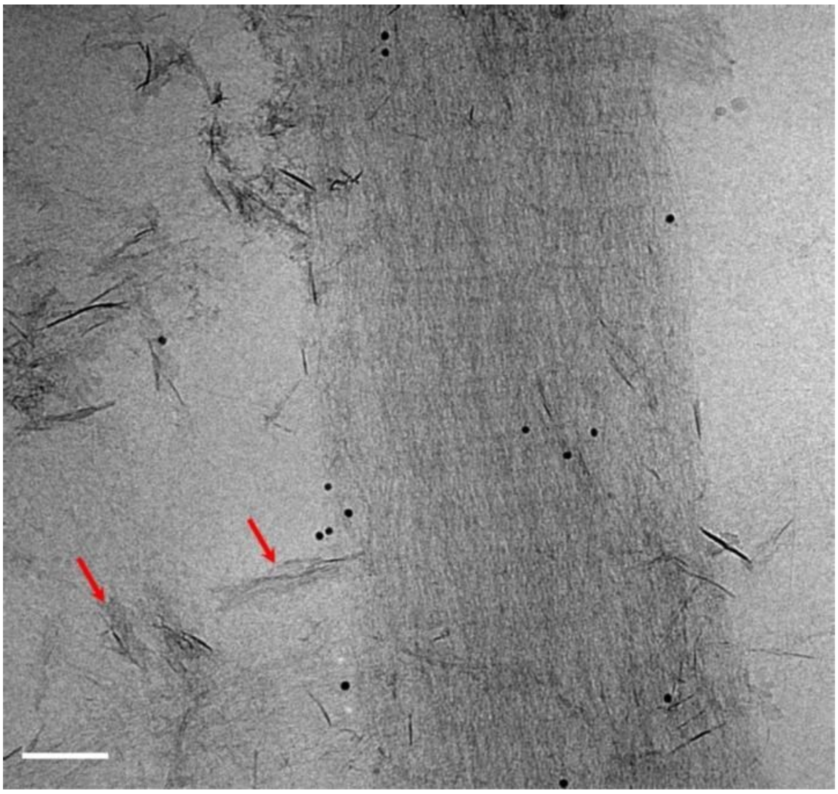

Figure 9. CryoTEM image of HAp crystals formed in solution. Arrows indicate representative crystals that were measured. Scale bar: 100 nm.

We measured the size of HAp crystals that formed outside collagen fibril. Supplementary Figure 9 shows a typical cryoTEM image, which was used to measure the size of the crystals. Only crystals found in the solution were measured, with length of ~130 nm, width of ~30 nm, thickness of ~4.5 nm and aspect ratio of ~4.3.

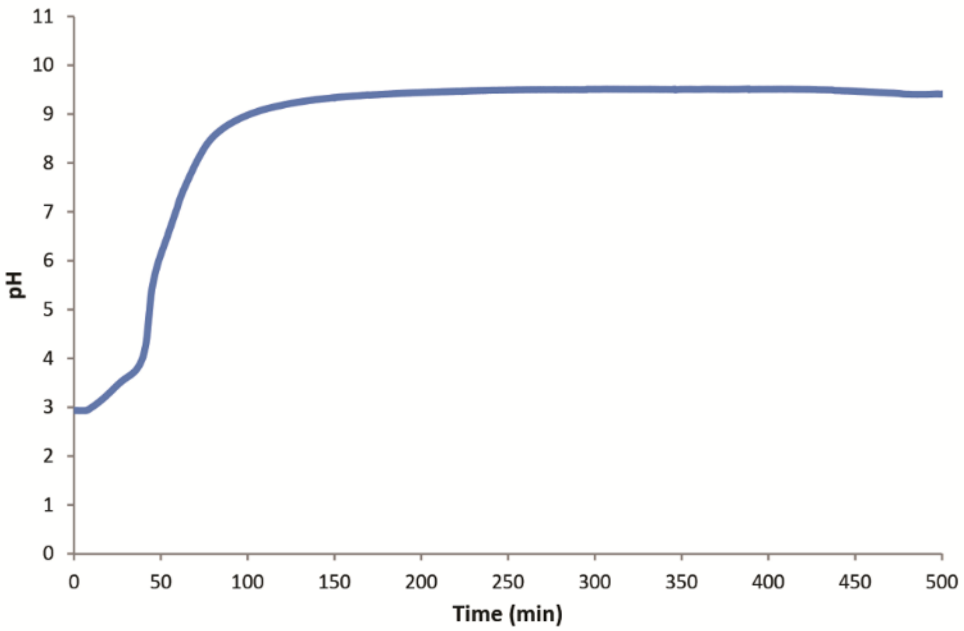

Figure 10. Increase of pH over time due to the diffusion of $NH_4(OH)$ 8% into a solution of Fe^{3+}/Fe^{2+} containing pAsp (Fe/Asp = 0.25).

Collagen sponges were placed in Fe^{3+}/Fe^{2+} solution containing pAsp (Fe/Asp = 0.25) and incubated overnight in a glove-box under N_2, saturated with 8% $NH_4(OH)$. Supplementary Figure 10 shows the increase in pH over time, as a result of the diffusion of $NH_4(OH)$ into the solution.

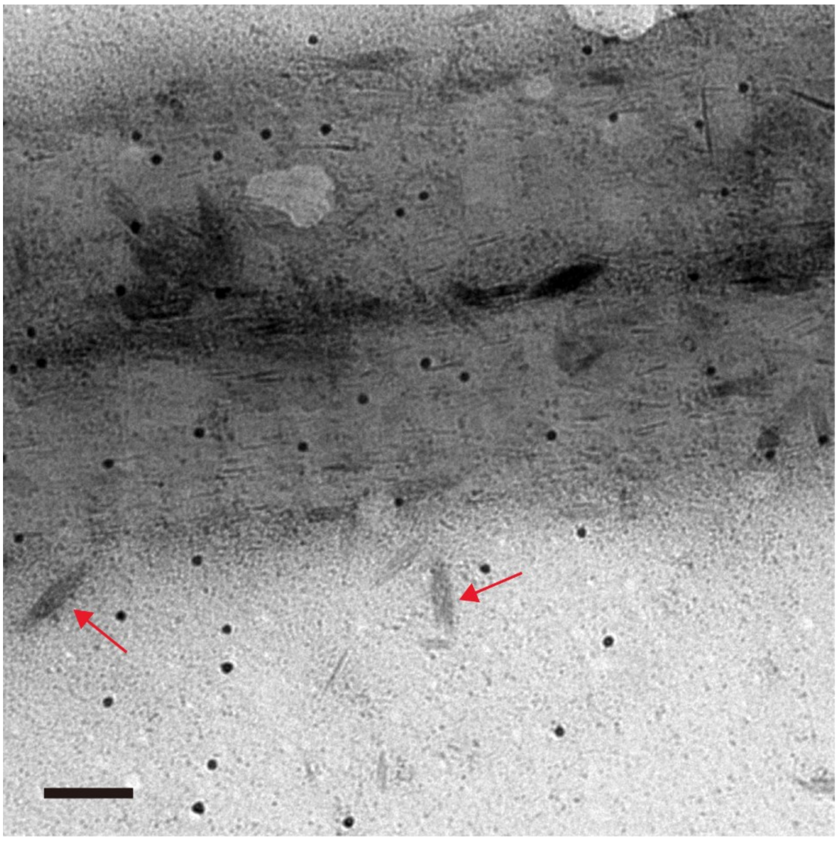

Figure 11. Dry TEM image of lepidocrocite crystals formed outside collagen fibril. Arrows indicate representative crystals that were measured. Scale bar: 100 nm.

We measured the size of lepidocrocite crystals that formed outside collagen fibril with a Fe: Asp ratio of 1:4. Supplementary Figure 11 shows a typical dry TEM image, which was used to measure the size of the crystals. Only crystals outside the fibril were measured, with length of ~77 nm, width of ~25 nm, thickness of ~3 nm and aspect ratio of ~3.1

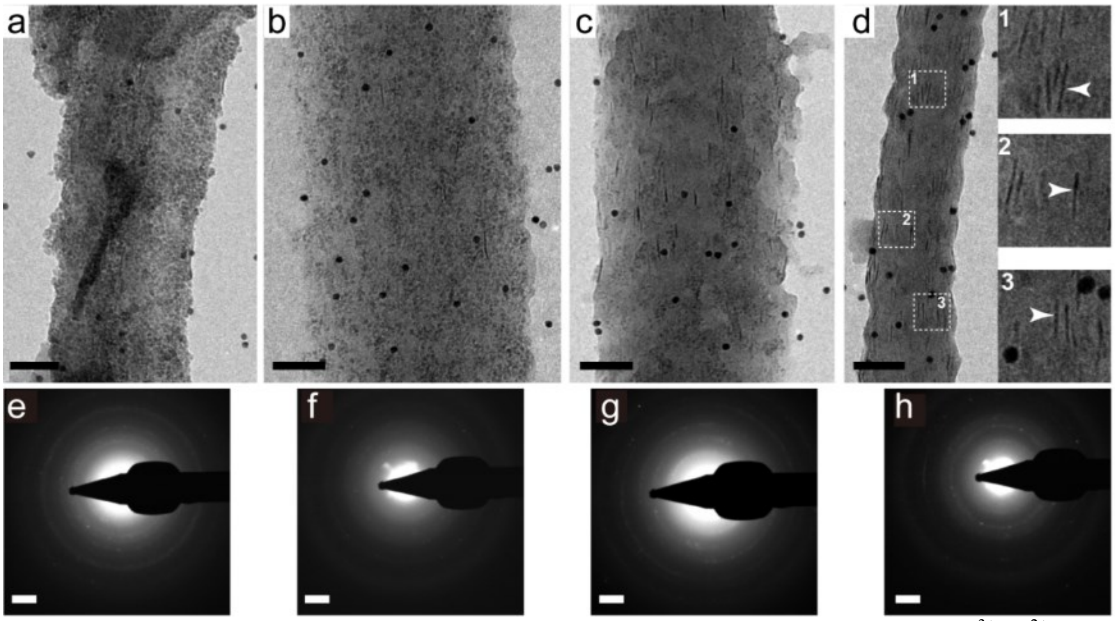

Figure 12. (a, c, e, g). Dry TEM image of collagen fibril mineralized by co-precipitation of Fe^{3+}/Fe^{2+} ions, with Fe:Asp ratio of 1:0, 4:1, 1:1 and 1:4, respectively. Insets of (g): Zoom-in images showing the lepidocrocite crystals aligned along the long axis of collagen (arrowheads). (b, d, f, h) LDSAED of (a, c, e, g), respectively. (b) and (d) correspond to 2-line ferrihydrite, while (f) and (h) shows the formation of lepidocrocite. Scale bars: (a, c, e, g) 100 nm. (b, d, f, h) 2 nm^{-1}.

When the mineralization was performed without any additives, we obtained collagen fibrils that were completely coated with ferrihydrite particles 5-10 nm in size (Supplementary Figures 12a and 12b). With a ratio of Fe: Asp of 4:1, the collagen was still covered with ferrihydrite, however few needle-shaped crystals were visible, with their long axis aligned in the direction of the long axis of the fibril (Supplementary Figures 12c and 12d). When the concentration of pAsp was increased to a ratio of 1:1 Fe: Asp, ferrihydrite did not form in the reaction anymore. Instead, lepidocrocite was present, both randomly oriented and with their long axis aligned in the direction of the collagen fibril (Supplementary Figures 12e and 12f). By increasing the concentration of pAsp to a ratio of 1:4 Fe: Asp, only oriented lepidocrocite crystals were visible (Supplementary Figures 12g and 12h).

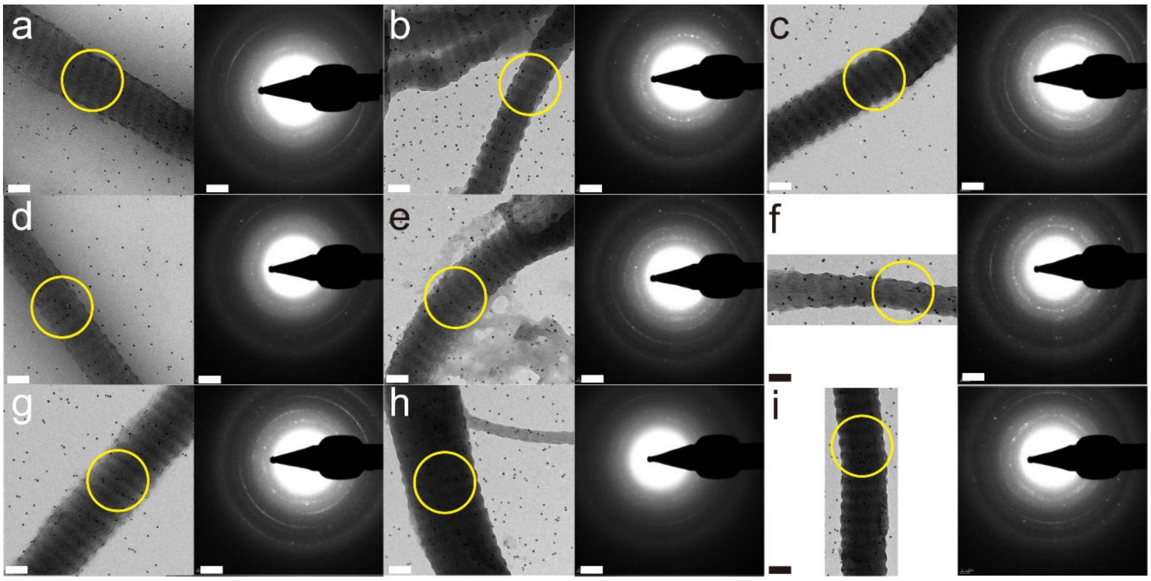

Figure 13. (a-i) Summary of 9 TEM images and corresponding LDSAED patterns of collagen mineralized with lepidocrocite in the presence of polyaspartic acid. The areas where the LDSAED patterns were taken are highlighted by yellow circles. Scale bars: TEM images: 100 nm. LDSAED patterns: 2 nm^{-1}.

The 9 LDSAED patterns (Supplementary Figure 13) were radially averaged. These 9 averages were again averaged to give a 1D diffraction spectrum as shown in Supplementary Figure 14. The peak positions and corresponding reflections are summarized in Supplementary Table 1. The reflections were assigned to lepidocrocite with the help of the XRD data reported by Ewing.[8]

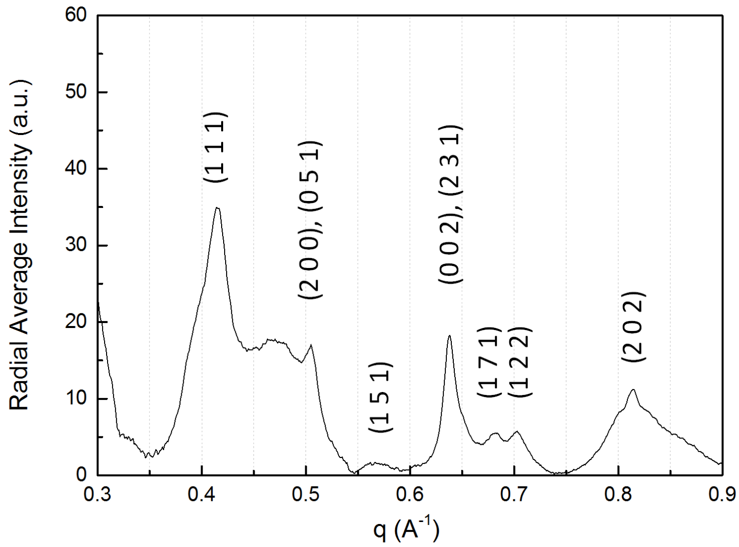

Figure 14. Radially averaged diffraction data from the average of all LDSAED patterns.

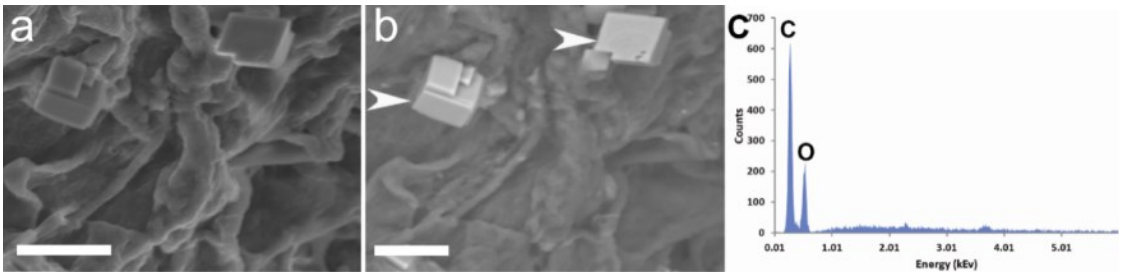

Figure 15. SEM images of the collagen sponge mineralized with $CaCO_3$ without the presence of polymeric additives. (a) Secondary electrons detector image of the collagen sponge. (b) Back-scattered electrons detector image of (a) White arrowheads: calcite crystals found between the collagen fibrils. (c) EDX analysis of (a), showing the absence of Ca in the collagen fibrils. Scale bars: 2 μm.

When the collagen sponges were mineralized without polymeric additives, rhombohedral calcite crystals were found between the collagen fibrils (Supplementary Figure 15a). Those crystals show a higher brightness in the back-scattered electron image comparing to the fibrils, indicating that no $CaCO_3$ was formed within the fibrils (Supplementary Figure 15b). This was confirmed by energy dispersive X-ray spectroscopy (EDX, Supplementary Figure 15c), which shows no Ca signal in the collagen fibrils.

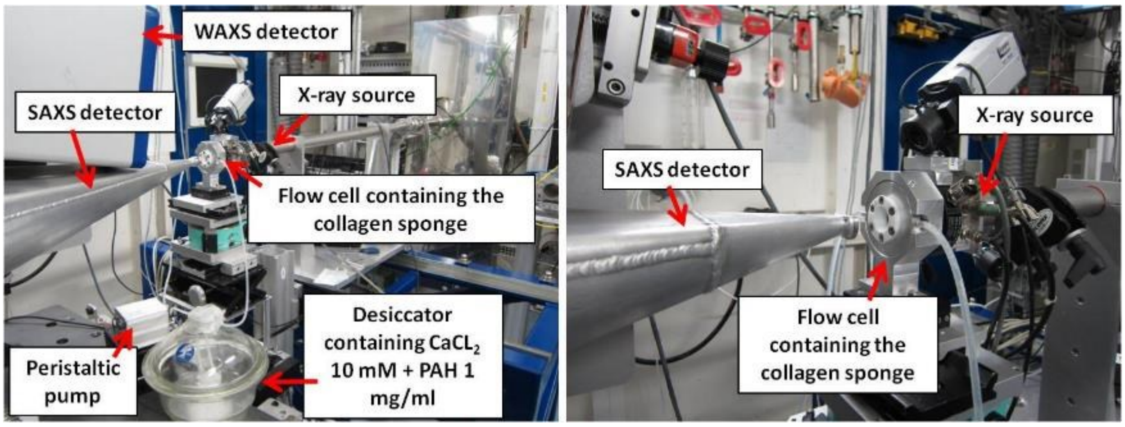

Figure 16. Experimental setup for measuring simultaneously SAXS and WAXS spectra during the mineralization of collagen sponges with $CaCO_3$ in the presence of pAH.

To perform in situ SAXS and WAXS study on the mineralization of collagen sponges with $CaCO_3$, 0.125 cm^3 of collagen sponge (bovine Achilles) was incubated in a flow cell between two mica windows. Subsequently, a solution of 10 mM $CaCl_2$ containing 1 mg mL^{-1} of pAH in a desiccator containing $(NH_4)_2CO_3$ was pumped through the flow cell with an addition rate of 2.5 ml/min. The flow cell was analyzed using SAXS/WAXS. The data were acquired every 5 minutes with an acquisition time of 3 minutes, over an 8 h period. The X-ray wavelength was set to $\lambda = 0.1$ nm and the sample-to-detector distances were 130 cm and 28 cm for SAXS and WAXS, respectively. The q-range (where $q = 4\pi/\lambda \sin(\theta)$ is the modulus of the scattering vector, and θ is half of the scattering angle) was calibrated according to the position of diffraction peaks from Silver Behenate and α-Alumina standard samples. High sensitivity, noiseless photon counting Pilatus detectors (Pilatus1M for SAXS and Pilatus 300K for WAXS) were used to collect 2D images. Standard corrections for beam intensity, empty cell background and sample transmission have been applied before 2D images integration.

Supplementary Tables

Reciprocal Distance ($Å^{-1}$)	Distance(Å)	Reflection
0.423	2.366	(1 1 1)
0.480	2.09	(0 6 0), (1 3 1)
0.516	1.94	(0 5 1)
0.517	1.93	**(2 0 0)**
0.578	1.729	(1 5 1)
0.652	1.534	**(0 0 2)**, (2 3 1)
0.696	1.436	(1 7 1), (1 8 0)
0.718	1.394	(1 2 2)
0.832	1.203	(2 0 2)

Table 1. Summary of the positions of diffraction peak maxima and corresponding reflections.

To generate the averaged diffraction pattern used in Figure 2c, the center of the individual diffraction patterns (Supplementary Figure 13) was determined by fitting a ring on the (002) wedge and the orientation was deduced from the corresponding TEM images. Subsequently, these diffraction patterns were translated and rotated with respect to their center and orientation resulting in aligned diffraction patterns. From these, the beamstop was removed by fitting a mask and the diffraction patterns were normalized with the average intensity in the 0.3-0.9 $Å^{-1}$ range. Next, the diffraction pattern was calculated and the background was subtracted with the imtophat function in Matlab using a disk of ~ 0.1 $Å^{-1}$ as structuring element. This yielded the final averaged diffraction pattern, with the all reflections aligned to give the collagen a vertical orientation, with respect to the averaged diffraction pattern.

Supplementary Notes

Note 1. Influential books/papers presenting the deck-of-cards model

Some of the influential books/papers presenting the deck-of-cards model of mineralized collagen fibril in bone are listed below together with the citation numbers. All the citation numbers are based on Google Scholar records on 20, Mar. 2020:

1. Lowenstam, H. A. and S. Weiner (1989). On biomineralization, Oxford University Press on Demand. (Cited: 3486)
2. Weiner, S. and H. D. Wagner (1998). "The material bone: Structure mechanical function relations." Annual Review of Materials Science **28**: 271-298. (Cited: 2660)
3. Rho, J.-Y., et al. (1998). "Mechanical properties and the hierarchical structure of bone." **20**(2): 92-102. (Cited: 2412)
4. Mann, S. (2001). Biomineralization: principles and concepts in bioinorganic materials chemistry, Oxford University Press on Demand. (Cited: 2709)
5. Dorozhkin, S. V. and M. J. A. C. I. E. Epple (2002). "Biological and medical significance of calcium phosphates." **41**(17): 3130-3146. (Cited: 1838)
6. Gao, H. J., et al. (2003). "Materials become insensitive to flaws at nanoscale: Lessons from nature." Proceedings of the National Academy of Sciences of the United States of America **100**(10): 5597-5600. (Cited: 1569)
7. Currey, J. D. (2006). Bones: structure and mechanics, Princeton university press. (Cited: 1957)
8. Fratzl, P. and R. Weinkamer (2007). "Nature's hierarchical materials." Progress in materials Science **52**(8): 1263-1334. (Cited: 1897)
9. Meyers, M. A., et al. (2008). "Biological materials: structure and mechanical properties." **53**(1): 1-206. (Cited: 1874)
10. Wegst, U. G., et al. (2015). "Bioinspired structural materials." **14**(1): 23-36. (Cited 1672)

Supplementary References

1 Landis, W. J., Hodgens, K. J., Arena, J., Song, M. J. & McEwen, B. F. Structural relations between collagen and mineral in bone as determined by high voltage electron microscopic tomography. *Microsc. Res. Tech.* **33**, 192-202 (1996).

2 Landis, W. J., Song, M. J., Leith, A., Mcewen, L. & Mcewen, B. F. Mineral and Organic Matrix Interaction in Normally Calcifying Tendon Visualized in 3 Dimensions by High-Voltage Electron-Microscopic Tomography and Graphic Image-Reconstruction. *J. Struct. Biol.* **110**, 39-54 (1993).

3 Friedrich, H., de Jongh, P. E., Verkleij, A. J. & de Jong, K. P. Electron tomography for heterogeneous catalysts and related nanostructured materials. *Chem. Rev.* **109**, 1613-1629 (2009).

4 Reznikov, N., Bilton, M., Lari, L., Stevens, M. M. & Kröger, R. Fractal-like hierarchical organization of bone begins at the nanoscale. *Science* **360**, eaao2189 (2018).

5 Cosslett, V. E. High voltage electron microscopy and its application in biology. Philosophical Transactions of the Royal Society of London. B, *Biol. Sci.* **261**, 35-44 (1971).

6 Nudelman, F. et al. The role of collagen in bone apatite formation in the presence of hydroxyapatite nucleation inhibitors. *Nat. Mater.* **9**, 1004-1009 (2010).

7 Olszta, M. J. et al. Bone structure and formation: A new perspective. *Mater. Sci. Eng. R.* **58**, 77-116, (2007).

8 Ewing, F. The crystal structure of lepidocrocite. *J. Chem. Phys.* **3**, 420-424 (1935).
