# Supplementary material for: Intermolecular channels direct crystal orientation in mineralized collagen
Source: Nat Commun. 2020 Oct 8;11:5068. doi: 10.1038/s41467-020-18846-2 (PMC7545172; doi:10.1038/s41467-020-18846-2)
Supplement: Supplementary file 3 — Description of Additional Supplementary Files [file 41467_2020_18846_MOESM3_ESM.pdf]

Description of Additional Supplementary Files

Supplementary Movie 1. Electron tomographic reconstruction y-slices of the human bone lamella, showing the overview of the sample.

Supplementary Movie 2. Electron tomographic reconstruction y-slices of the two mineralized collagen fibrils in human bone as shown in Figures 2a to 2h.

Supplementary Movie 3. Electron tomographic reconstruction z-slices of the two mineralized collagen fibrils in human bone as shown in Figures 2a to 2h.

Supplementary Movie 4. 3D electron density map of collagen structure created from Xray diffraction data. The white fibrils correspond to the collagen molecules, and the channels in the gap region are labelled by different colors based on their connectivity.

Supplementary Movie 5. CryoEM tomographic reconstruction y-slices of a collagen fibril mineralized in vitro by HAp as shown in Figures 2i to 2p.

Supplementary Movie 6. CryoEM tomographic reconstruction z-slices of a collagen fibril mineralized in vitro by HAp as shown in Figures 2i to 2p.

Supplementary Movie 7. EM tomographic reconstruction y-slices of a collagen fibril mineralized in vitro by lepidocrocite as shown in Figure 4.

Supplementary Movie 8. EM tomographic reconstruction z-slices of a collagen fibril mineralized in vitro by lepidocrocite as shown in Figure 4.
